# Liver expression quantitative trait loci: a foundation for pharmacogenomic research

**DOI:** 10.3389/fgene.2012.00153

**Published:** 2012-08-14

**Authors:** Dylan M. Glubb, Neepa Dholakia, Federico Innocenti

**Affiliations:** ^1^Institute for Pharmacogenomics and Individualized Therapy, University of North Carolina at Chapel HillChapel Hill, NC, USA; ^2^Eshelman School of Pharmacy, University of North Carolina at Chapel HillChapel Hill, NC, USA; ^3^Lineberger Comprehensive Cancer Center, University of North Carolina at Chapel HillChapel Hill, NC, USA

**Keywords:** liver, expression quantitative trait loci, ADME genes, GWAS, clinical pharmacogenomics

## Abstract

Expression quantitative trait loci (eQTL) analysis can provide insights into the genetic regulation of gene expression at a genomic level and this information is proving extremely useful in many different areas of research. As a consequence of the role of the liver in drug metabolism and disposition, the study of eQTLs in primary human liver tissue could provide a foundation for pharmacogenomics. Thus far, four genome-wide eQTL studies have been performed using human livers. Many liver eQTLs have been found to be reproducible and a proportion of these may be specific to the liver. Already these data have been used to interpret and inform clinic genome-wide association studies, providing potential mechanistic evidence for clinical associations and identifying genes which may impact clinical phenotypes. However, the utility of liver eQTL data has not yet been fully explored or realized in pharmacogenomics. As further liver eQTL research is undertaken, the genetic regulation of gene expression will become much better characterized and this knowledge will create a rational basis for the prospective pharmacogenomic study of many drugs.

## Introduction

As research has progressed, so has our understanding of how the genome plays a role in drug disposition and response via genes related to drug targets or absorption, distribution, metabolism, and excretion (ADME) (Goldstein et al., 2007). Indeed, germline human genetic variation is well known to affect drug disposition and clinical responses to pharmacotherapy. The Pharmacogenomics Knowledgebase (PharmGKB; www.pharmgkb.org) curates pharmacogenetic findings from the literature and, as of May 2012, lists 411 associations between germline genetic variants and drug-related phenotypes. Only 20% of these associations can be explained by genetic variation which leads to distinct protein differences through altered amino acid sequences. However, genetic variation can impact ADME gene expression in many different ways, at either mRNA or protein level (Glubb and Innocenti, 2011). Moreover, transcript levels provide clearly defined and measurable traits which may act as intermediates for drug-related phenotypes.

Genetic variants which associate with gene expression are known as expression quantitative trait loci (eQTLs) and can be further characterized as *cis* or *trans*. *cis*-eQTLs are located near the expressed gene, usually defined by an arbitrary distance (Gilad et al., 2008), whilst *trans*-eQTLs are located further away, sometimes on a different chromosome to the expressed gene. eQTLs can refer to any type of genetic variation but most often indicate that a single nucleotide polymorphism (SNP) genotype is associated with the transcript level of a gene. The interrogation of SNP genotypes and transcript levels have generally been performed using oligonucleotide microarray platforms, enabling the identification of eQTLs through genome-wide analyses (Franke and Jansen, 2009). This linking of gene variation and genetic expression may clarify the pharmacogenetic significance of the gene of interest (Li and Deng, 2010) and, thus, eQTLs have significant potential utility for the study of pharmacogenomics.

Lymphoblastoid cell lines (LCLs) have been the predominant model for eQTL analyses in humans (Skelly et al., 2009). These cell lines are derived from B-lymphocytes transformed by the Epstein-Barr virus (EBV). Although this transformation provides an immortalized cell line for study, which can be directly examined in pharmacogenomic studies (Huang et al., 2007a,b; 2008), there are limitations to their use as non-genetic factors can affect gene expression (Choy et al., 2008). These factors include the life history of the human donor, the B-lymphocyte subtype selected, the EBV titers used for transformation, the culture conditions in which the LCLs are grown and the intrinsic characteristics of the cell line. Nevertheless, several studies have translated LCL eQTL findings in clinical pharmacogenomic studies [reviewed in (Wheeler and Dolan, 2012)].

In the context of clinical pharmacogenomics, the liver may be the most relevant tissue in which to perform eQTL analyses. The liver is the predominant organ in drug elimination, with 75% of the 200 most widely prescribed drugs being eliminated from the body through liver metabolism or biliary excretion (Wienkers and Heath, 2005). Furthermore, a significant number of ADME genes are strongly expressed in the liver. Schroder et al. defined a list of 682 ADME genes from various pharmacogenomic resources (Schroder et al., 2011) and the expression of 61 of these genes have been predicted to be liver-specific according to a study of gene expression in human tissues (Yu et al., 2006). Therefore, eQTLs of these genes may not be detected in other tissues. In addition, eQTLs may have effects at the molecular level that are dependent upon the cell or organ in question. In this review, the first to our knowledge to focus specifically on liver eQTLs, we aim to show that the liver eQTL knowledge could provide a foundation to clinical pharmacogenomic research.

## Liver eQTL studies

We are presently in the early stages of liver eQTL research and only four genome-wide eQTL studies have been reported which describe the analysis of primary human liver tissue (Table 1). However, several thousand eQTLs have been identified after correction for multiple testing and many of the eQTL SNPs (eSNPs) associate with the expression of ADME genes.

**Table 1:** **Summary of human liver eQTL studies**.

**Category**	**Schadt et al.**	**Innocenti et al.**	**Schroder et al.**	**Greenawalt et al.**
Samples in final analysis (n)	384	206	149	651
Race	All Caucasian	183 European American 23 African American	All Caucasian	86% of overall cohort Caucasian
Nature of liver tissue	Postmortem tissue and resections from donor livers	Postmortem tissue and resections from donor livers	Normal tissue resected during surgery for liver cancer	Tissue from morbidly obese patients undergoing gastric bypass
Covariates	Age, sex and medical center	Age, sex, genetic ancestry, effects of array hybridization and oligonucleotide probes, and surrogate variables	Age, sex, smoking, alcohol consumption, diagnosis, C-reactive protein, cholestatic liver disease and presurgical medication	Age, sex and medical center
Genotyping platform (GEO dataset)	Affymetrix 500 k Illumina 650Y (GSE# n/a)	Illumina 610 Quad (GSE26105)	Illumina HumanHap300 (GSE39036)	Illumina 650Y (GSE# n/a)
Expression platform (GEO dataset)	Agilent Custom 44k (GSE9588)	Agilent 4×44k (GSE25935)	Illumina HumanWG-6 v2.0 (GSE32504)	Agilent Custom 44k (GSE24293)
*cis*-eQTLs (n)	1350 (Bonferroni adjusted) 3210 (FDR<10%)	1787 (BF>5)	1179 (Bonferroni adjustment)	7902 (FDR<10%)
*trans*-eQTLs (n)	242 (Bonferroni adjustment) 491 (FDR<10%)	353 (BF>5)	47 (Bonferroni adjustment)	785 (FDR<10%)

### Liver eQTLS in pharmacogenetic studies and clinical genome-wide association studies (GWAS)

ADME genes with liver eQTLs, which pass multiple correction thresholds in one of the four genome-wide studies, include *COMT*, *CYP2D6*, *CYP3A4*, *CYP3A5*, *MTHFR*, *UGT1A1*, and *VKORC1* (Table 2). These genes belong to a set of ‘very important pharmacogenes’ which have been identified by the PharmGKB resource (http://www.pharmgkb.org/search/browseVip.action?browseKey=vipGenes). Furthermore, several of these eSNPs are markers of pharmacogenetic associations (Table 2). These findings show that liver eQTLs may help explain the associations of SNPs with clinical phenotypes and also provide a rationale for using liver eQTL data from relevant genes to select candidate SNPs for pharmacogenomic study. For example, the three eSNPs identified in the *CYP2D6* gene (Table 2) could conceivably be used to test associations with pertinent clinical phenotypes of any of the 46 drugs that, according to PharmGKB curated information (http://www.pharmgkb.org/gene/PA128#tabview=tab6&subtab=21), are metabolized by the CYP2D6 enzyme.

**Table 2:** **Liver eQTLs of PharmGKB ‘very important pharmacogenes’^a^**.

**Pharmacogene**	**eSNP**	**eQTL study**	**PharmGKB pharmacogenetic SNPs^b^ sharing perfect LD^c^ (*r*^2^ = 1) with liver eSNP**	
			**Pharmacogenetic SNP**	**Related drug(s)**
*COMT*	rs174674	Schadt et al.	−	−
*CYP2D6*	rs151096	Schadt et al.	−	−
	rs8138080	Schadt et al.	−	−
	rs133337	Innocenti et al.	−	−
*CYP3A4*	rs7792939	Greenawalt et al.	−	−
*CYP3A5*	rs10242455	Schadt et al. Schroder et al.	rs4646457 rs776746	Tacrolimus Cyclosporine Nevirapine Paclitaxel Rosuvastatin Sirolimus Sunitinib Tacrolimus
	rs1859690	Schadt et al. Schroder et al.	rs4646457 rs776746	Tacrolimus Tacrolimus Cyclosporine Nevirapine Paclitaxel Rosuvastatin Sirolimus Sunitinib
*MTHFR*	rs1801131	Innocenti et al.	rs1801131	Methotrexate Nitrous oxide
*UGT1A1*	rs2070959	Schroder et al.	−	–
*VKORC1*	rs2303222	Innocenti et al.	rs2359612 rs8050894	Warfarin Warfarin
	rs10871454	Schroder et al.	rs2359612 rs8050894	Warfarin Warfarin
	rs889548	Schroder et al.	−	−
	rs749767	Schroder et al.	−	−
	rs4889606	Schadt et al.	−	−

a PharmGKB has currently identified 46 genes of particular importance in pharmacogenomics (http://www.pharmgkb.org/search/browseVip.action?browseKey=vipGenes).

b From the PharmGKB curated list of level 1–3 SNP-drug clinical annotations (http://www.pharmgkb.org/search/clinicalAnnotationList.action?evidenceStrength=1).

c LD determined from 1000 Genomes Pilot 1 CEU data using SNAP browser (http://www.broadinstitute.org/mpg/snap/ldsearch.php).

Liver eQTL data can also be used to inform GWAS. GWAS have identified many SNPs which are associated with clinical traits in humans. Typically there is no mechanistic explanation for the association but eQTLs could help fill this gap in the knowledge. Amongst the liver eQTL studies, Greenawalt et al. identified 122 eSNPs which were significantly associated, after multiple test correction, with a clinical phenotype in a GWAS (Greenawalt et al., 2011) and we found 32 eSNPs that were significant GWAS SNPs or markers for a GWAS SNP through strong linkage disequilibrium (LD) (*r*^2^ > 0.8) (Innocenti et al., 2011). In fact, there is a significant enrichment of eSNPs among SNPs associated with clinical phenotypes in GWAS (Nicolae et al., 2010). However, a critical piece of information which is lacking from these reports is the directionality of the eQTL association (i.e., which allele associates with higher/lower transcript levels) and, thus, hypotheses about the effect of a specific allele cannot be generated.

Another use for eQTLs is in determining which gene a SNP, associated with a clinical phenotype, may mediate its effect through as there is often uncertainty about GWAS SNPs located in intergenic regions. Schadt et al. provide examples of intergenic eSNPs that were found to associate with type 1 diabetes, coronary artery disease and LDL cholesterol levels in GWAS (Schadt et al., 2008). By identifying the gene whose expression the GWAS SNP associates with, evidence is provided to suggest that the gene may be a susceptibility factor for the disease.

### Liver eQTL reproducibility

There are many factors related to the liver eQTL studies which could act as confounders and affect eQTL reproducibility. Firstly, the liver eQTL studies use a variety of tissue sources from individuals with different ethnic backgrounds (Table 1). Tissue sources include resections of normal tissue from patients suffering from liver cancer (Schroder et al., 2011) or morbid obesity (Greenawalt et al., 2011). Resections of tissue from healthy and deceased liver donors, who may have undergone multiple pharmacotherapeutic treatments immediately prior to death, have also been used (Schadt et al., 2008; Innocenti et al., 2011). Secondly, different genotyping and gene expression platforms, and analysis methods were used to generate eQTLs (Table 1). Furthermore, these studies control for different covariates and have variable powering due to the number of samples, genotypes and transcripts interrogated (Table 1). Despite these issues, there is evidence that a substantial proportion of the *cis*-eQTL findings are reproducible. In our liver eQTL study, we attempted to replicate eQTL findings in two additional datasets, one of which was a subset of samples from the Schadt et al. study (Innocenti et al., 2011). 67% of the significant *cis*-eQTLs (Bayes factor (BF)>5) replicated in at least one of the additional datasets (*p* < 0.05 and concordant effect direction), but this replication rate dropped to 6% for *trans*-eQTLs (BF>5) (Innocenti et al., 2011). Greenawalt et al. examined the reproducibility of their liver *cis*-eQTLs (FDR<10%) in the Schadt et al. study, which had used the same microarray gene expression platform, and found a 66% overlap (Greenawalt et al., 2011). Using Bonferroni adjusted *p*-values, a much more stringent analysis, a comparison between the Schadt et al. and Schroder et al. studies showed that only 16% of *cis*-eQTLs replicated and no replication of *trans*-eQTLs (Schroder et al., 2011). Neither of the latter two studies state whether a concordant effect direction was a criterion for replication but it appears that liver *cis*-eQTLs are comparatively reproducible whilst *trans*-eQTLs are not. This observation may be partly explained by statistical power. Compared with *cis*-eQTLs, *trans*-eQTLs have a greater multiple test correction burden and, thus, a *trans*-eQTL is less likely to achieve statistical significance in two independent studies.

A salient finding in our study was that the eSNP with the highest BF was frequently located immediately upstream or downstream of the transcription start site (TSS) of the eQTL gene. Our group discovered that 75% of *cis*-eQTLs within 100 kb of the TSS were found to replicate, compared to 61% of those located more than 100 kb from the TSS (Innocenti et al., 2011). Furthermore, Schadt et al. noted in their study that 70% of the *cis*-eQTLs were found within 100 kb of the TSS (Schadt et al., 2008). Therefore, in general, it appears that reproducible liver *cis*-eQTL SNPs will be found close to gene TSSs. This suggests that these eSNPs may directly impact gene transcription by altering gene regulatory regions.

### Liver eQTL tissue specificity

To examine tissue specificity of liver eQTLs, Schadt et al. compared their findings with eQTL analyses of human blood and adipose tissue (Emilsson et al., 2008). Approximately 30% of the *cis*-eQTLs identified in these tissues were also detected among the liver *cis*-eQTLs (FDR<10%) from their study (Schadt et al., 2008). Greenawalt et al., examined replication of their liver *cis*-eQTLs in adipose tissue from the same cohort of individuals used for liver eQTL analysis and found 46–48% of the liver *cis*-eQTLs replicated at *p* < 0.05 (Greenawalt et al., 2011). These observations suggest that some fraction of the genetic control of gene expression is specific to the liver. Indeed, Greenawalt et al. found that liver eQTLs, but not eQTLs identified from adipose tissue, were enriched for the KEGG pathway *drug metabolism-cytochrome P450 genes*. Furthermore, of the 11 ADME genes which had reproducible eQTLs between the Schroder et al. and Schadt et al. studies (Schadt et al., 2008; Schroder et al., 2011), *CYP3A5*, *DHRS2* and *SLC22A3* have been predicted to have liver-specific expression (Yu et al., 2006).

### Correlation of liver eQTLS and P450 enzyme activity

Gene transcript levels are a useful intermediate phenotype to study but measurements of protein levels or enzyme activity may provide a more informative phenotype for pharmacogenomics. One of the advantages of the liver eQTL model is that subcellular fractions can be generated from the liver, allowing proteomic and biochemistry studies to be integrated with genomic and transcriptomic data from the same tissue. For example, Yang et al. examined the enzyme activity of eight P450 proteins using liver microsomal samples derived from the same cohort as Schadt et al. (2008), and found that the activities of the CYP450s were positively correlated with mRNA transcript levels after adjusting transcript and activity levels for age, sex and study site (Yang et al., 2010). Transcript levels explained 10–30% of the variance in enzyme activity, thus, indicating that other biological factors, such as post-translational mechanisms, may also impact on activity levels. Fifty four enzyme activity QTLs (aQTLs) were identified for the eight P450 proteins: 24 SNPs were *trans*-aQTLs and 30 *cis*-aQTLs (FDR<10%) (Yang et al., 2010). The *cis*-aQTL SNPs were all associated with CYP2D6 enzyme activity and 29 of these were found to be also *cis*-eSNPs of *CYP2D6* (FDR<10%).

### Web-based liver eQTL data resources

The Gene Expression Omnibus (GEO) repository contains data from all the human liver eQTL studies (Table 1), although genotypes from the Schadt et al. and Greenawalt et al. studies are not available. The data cannot be easily interrogated and, thus, we plan to upload the liver eQTL findings of our study to the web-based SNP and CNV Association (SCAN) database (Gamazon et al., 2010). The results of the Schadt et al. study are already available through the Genotype-Tissue Expression (GTEx) eQTL browser (http://www.ncbi.nlm.nih.gov/gtex/GTEX2/gtex.cgi), and can be queried by gene or SNP and filtered by *p*-value or *r*^2^. The GTEx browser provides further useful information by annotating corresponding eSNPs with significant findings from GWAS.

## Future directions

### Cnvs

Single nucleotide base changes are the most common but not the only germline genetic variation present in the human genome. A study of genetic variation in eight individuals identified four million SNPs, ~800,000 insertion/deletions (>1 – 100 bp) and 1695 structural (>6 kb) variants, also known as copy number variants (CNVs) (Kidd et al., 2008). Although CNVs are much fewer in number than SNPs, their potential to impact on gene expression is greater due to their size and ability to increase gene copy number or ablate the transcriptional unit. Indeed, SNPs which are in strong LD (*r*^2^ = 0.8) with CNVs are more likely to be LCL eQTLs and associated with the expression of more than one gene (Gamazon et al., 2011). Moreover, the importance of CNVs in pharmacogenetics is already well known. CNVs with functional effects on enzyme activity of proteins (e.g., CYP2D6, GSTM1 and GSTT1) have been observed to associate with drug efficacy and toxicity (He et al., 2011). It is likely that these CNVs may be eQTLs and, thus, there is a great need for liver CNV eQTL analyses to be carried out in the future.

### Next-generation sequencing

Previous liver eQTL studies have interrogated genetic variation and gene expression at the genome level. However, due to the limitations of the oligonucleotide genotyping and gene expression microarray platforms, this corresponds only to a fraction of human genetic variation and does not encompass all transcript splice variants or gene isoforms.

Genotype imputation can be performed using genotype data from studies like HapMap or 1000 Genomes Project to greatly increase the number of genotypes interrogated through oligonucleotide genotyping platforms. Indeed, in our liver eQTL study we imputed genotypes from HapMap to generate more than two million genotypes (Innocenti et al., 2011). While still relatively expensive, next-generation sequencing has advanced to the point where it is becoming feasible to perform whole genome sequencing of DNA and mRNA to comprehensively characterize genetic variation and the entire transcriptome in a relatively large number of samples.

Although there is high correlation between transcript levels quantitated by RNA-seq and microarrays (Pickrell et al., 2010), RNA-seq provides a greater resolution which leads to detection of more eQTLs (Montgomery et al., 2010). The RNA-seq (next generation sequencing of mRNA) approach has other advantages over oligonucleotide expression arrays e.g., no saturation of intensity signal for high abundance transcripts and the ability to quantify allele-specific expression (Majewski and Pastinen, 2011). Additionally, RNA-seq can identify splicing QTLs which may not affect overall gene expression but alter transcript isoform levels (Pickrell et al., 2010).

### Identification of functional eSNPs

eQTL analyses are informative but they cannot definitively identify the causative SNPs that affect gene expression. Due to LD, many eSNPs can represent the same causative genetic variant. Various approaches can be used to identify functional genetic variants which generate the eQTL signal. Reporter gene assays are useful in determining whether eSNPs in promoter, 5′UTR, 3′UTR and intronic regions have functional effects on gene expression (Innocenti et al., 2011). However, this technique cannot be easily applied in a high-throughput fashion and the effects of SNPs in coding regions are generally not amenable to testing.

Bioinformatic analyses may help to narrow the number of putatively causative SNPs, by identifying eSNPs which are located in functional genomic elements such as those defined by the Encyclopedia of DNA Elements (ENCODE) Project (Myers et al., 2011). Indeed, a study of LCL eQTLs found that 40% of the eSNPs were located in open chromatin regions and sites of DNaseI hypersensitivity or histone modification. Furthermore, SNPs in DNaseI hypersensitivity sites were four-fold more likely to associate with transcript levels compared with other SNPs (Gaffney et al., 2012). This epigenetic, and other regulatory, information can be combined with eQTL data to create a model which ranks eSNPs on their potential to affect gene expression (Gaffney et al., 2012). This approach has not yet been applied to a study of liver eQTLs but could prove extremely useful in identifying pharmacogenetic variants.

### Epistatic eQTL interactions

eQTL analyses generally examine only the effects of a single genetic locus on gene expression. However, it is possible that interactions of *cis*- and *trans*-eQTLs contribute to the regulation of gene expression. Indeed, 15% of transcripts were found to be regulated by a *cis*-*trans* eQTL interaction in a study of HapMap LCLs and the vast majority of the eSNPs were not significantly associated with gene expression in a single locus analysis (Becker et al., 2012). Therefore, important information may be lost by not considering these eQTL interactions and this approach could be used to detect epistatic pharmacogenetic variants which may not otherwise be identified.

### Liver protein QTLs

The Yang et al. study (Yang et al., 2010) correlating liver eQTLs and P450 enzyme activity highlights the need for liver protein QTL analyses to characterize the genetic regulation of protein expression. High-throughput proteome-wide quantification of protein expression is much more problematic and less straightforward than that for transcriptome quantification. Liquid chromatography-tandem mass spectrometry (LC-MS/MS) techniques are providing a way forward but, currently, the best approach for sensitive and accurate protein quantitation may be to carry out targeted proteomic studies (Kline et al., 2009). For example, targeted LC-MS/MS analysis of several UGT1A isoforms has been successfully performed in human liver microsomes (Harbourt et al., 2012).

## Conclusion

There is a strong rationale for using findings from human liver eQTL studies as a knowledge base to inform and guide clinical pharmacogenomic studies. Liver eQTL analyses provide insights into the genetic regulation of ADME genes which may not be obtained from other tissues and identifies eQTLs which are reproducible and unique to the liver. Moreover, liver eQTL data can help explain previously identified pharmacogenomic associations and provides candidates for prospective clinical study. In the future, the integration of epistatic, epigenetic, CNV, next-generation sequencing, bioinformatic, and protein analyses will generate a truly comprehensive map of the genetic regulation of gene expression in the liver which is certain to profoundly impact pharmacogenomics.

### Conflict of interest statement

The authors declare that the research was conducted in the absence of any commercial or financial relationships that could be construed as a potential conflict of interest.
